# Active health Ombudsman service: evaluation of the quality of delivery and birth care

**DOI:** 10.11606/S1518-8787.2018052017291

**Published:** 2018-07-13

**Authors:** Karlo Jozefo Quadros de Almeida, Francis Nakle de Roure, Roberto José Bittencourt, Regina Maria Dias Buani dos Santos, Fernanda Viana Bittencourt, Leila Bernarda Donato Gottems, Fábio Ferreira Amorim

**Affiliations:** I Escola Superior de Ciências da Saúde. Coordenação de Pós-Graduação e Extensão Brasília, DF, Brasil; II Secretaria de Estado de Saúde do Distrito Federal. Ouvidoria em Saúde. Brasília, DF, Brasil; III Fundação de Ensino e Pesquisa em Ciências da Saúde. Coordenação de Projetos Estratégicos. Brasília, DF, Brasil

**Keywords:** Pregnant Women, Patient Advocacy, Patient Rights, Maternal-Child Health Services, Quality of Health Care, Gestantes, Defesa do Paciente, Direitos do Paciente, Serviços de Saúde Materno-Infantil, Qualidade da Assistência à Saúde

## Abstract

**OBJECTIVE:**

To evaluate the active health Ombudsman service as an instrument to evaluate the quality of delivery and birth care in the *Cegonha* Network of the Federal District of Brazil.

**METHODS:**

This is a cross-sectional study of the telephone survey type carried out with 1,007 mothers with deliveries between October 15, 2013 and November 19, 2013 in the twelve public maternity hospitals that make up the *Cegonha* Network of the Federal District of Brazil. The instrument has 25 multiple choice or Likert scale questions, including sociodemographic data and acceptability evaluation in five domains: accessibility, relationship between the patient and health professionals, conditions of the structure of the service, information to the patient, and equity and opinion of the patient. We have studied qualitative or categorical variables according to the frequency and distribution of proportions. We have used the score transformed into a scale from zero to 100 for the analysis of the Likert-type scale questions. Results have been expressed as mean and standard deviation.

**RESULTS:**

Access to prenatal appointments was evaluated as good or excellent by 86.1% of the participants and laboratory tests was evaluated as good or excellent by 85.2% of them. The access to imaging tests was evaluations as good or excellent by 45.7% of the women; 79.5% of the interviewees had their delivery in the maternity hospital where they sought initial care and 18.3% received a home visit by a community health agent after discharge. Most women reported that newborns were placed skin-to-skin immediately after birth, 48.9% had a companion at the time of the delivery, 76.3% were advised about the first appointment of the newborn, and 94.8% were advised on breastfeeding in the maternity hospital. Regarding the evaluation of health professionals, 85.9% of the women considered reception and cordiality as good or excellent at the prenatal care and 94.8% considered it as good or excellent at the maternity hospital.

**CONCLUSIONS:**

The active health Ombudsman service has contributed to evaluate the quality of public management by allowing the incorporation of the perspective of users of the health service in the evaluation of the acceptability of the *Cegonha* Network in the Federal District of Brazil.

## RESUMO

**OBJETIVO:**

Avaliar a ouvidoria ativa em saúde como ferramenta de avaliação da qualidade da atenção ao parto e nascimento da Rede Cegonha do Distrito Federal.

**MÉTODOS:**

Estudo transversal, do tipo inquérito por via telefônica, em 1.007 puérperas com partos realizados entre 15/10/2013 e 19/11/2013, nas 12 maternidades públicas que compõem a Rede Cegonha no Distrito Federal. O instrumento continha 25 questões de múltipla escolha ou do tipo escala *Likert*, que incluiu dados sociodemográficos e avaliação da aceitabilidade em cinco domínios: acessibilidade, relacionamento entre o paciente e os profissionais de saúde, condições da estrutura do serviço, informação ao paciente, e equidade e opinião do paciente. Variáveis qualitativas ou categóricas foram estudadas de acordo com a frequência e distribuição de proporções. Utilizou-se o escore transformado para uma escala de zero a 100 para análise das questões do tipo escala *Likert*. Resultados foram expressos em média e desvio-padrão.

**RESULTADOS:**

O acesso às consultas de pré-natal foi avaliado como bom ou ótimo por 86,1% das participantes e os exames laboratoriais como bom ou ótimo por 85,2%. O acesso aos exames de imagem teve 45,7% avaliações boas ou ótimas; 79,5% das entrevistadas realizaram o parto na maternidade onde buscaram atendimento inicial e 18,3% receberam visita domiciliar por agente comunitário de saúde após a alta. A maioria relatou que os recém-nascidos foram colocados em seus colos ou peitos imediatamente após o nascimento, 48,9% tiveram presença de acompanhante no momento do parto, 76,3% foram orientadas em relação à primeira consulta dos recém-nascidos e 94,8% sobre aleitamento materno na maternidade. Quanto à avaliação dos profissionais de saúde, 85,9% das mulheres consideraram o acolhimento e a cordialidade recebidos como bons ou ótimos no pré-natal e 94,8%, como bons ou ótimos na maternidade.

**CONCLUSÕES:**

A ouvidoria ativa em saúde contribuiu para a avaliação da qualidade da gestão pública, possibilitando a incorporação da perspectiva das usuárias do serviço de saúde na avaliação da aceitabilidade da Rede Cegonha no Distrito Federal.

## INTRODUCTION

Since the 10th Brazilian Health Conference in 1996, the Health Ombudsman service seeks to consolidate its institutional role within the scope of the Brazilian Unified Health System (SUS). After 20 years, there is no critical review on the actions taken by Health Ombudsman services or on their impacts on management^1^. The expectations regarding the performance of the Health Ombudsman service are great, in particular, as to the symbolism of advancing the participatory democracy established by a direct communication path between the population and the SUS management^2^. However, the academic production on this subject is incipient^1^.

Health Ombudsmen services have been active in promoting active spaces to approach the user in order to collect their demands and seek their opinions about the services. This is the active health ombudsman service (OAS). These services stop acting only passively, when they expect users to search for their demands, and they adopt ways to actively seek information. One of these ways is the application of research questionnaires that seek to know the reality and the quality of the health care of the users of the service to subsidize the management and social control. The OAS can be a powerful instrument to evaluate health services when promoting the exercise of direct democracy and research on user satisfaction as a strategy for social approximation and empowerment^2^ ^,^ ^3^.

The user of any health care system has the right to receive quality health care. In this way, public management must act to ensure the full quality of the care in the SUS, which is not yet feasible according to the constitutional precepts^3^ ^,^ ^4^. According to Donabedian^5^, good quality in health care is the one that provides the maximum well-being to the patient after considering the best balance between the gains and losses that follow the entire care process, in order to obtain the best results possible with the current scientific level. It consists of attributes that include efficiency, effectiveness, optimization, acceptability, legitimacy, and equity^5^ ^,^ ^6^.

Acceptability is the ability of the health system to satisfy the desires, wishes, and expectations of patients and their families, i.e., how the provision of health services is perceived by the population. The evaluation of the acceptability of a health system has five sub-dimensions: (1) accessibility: ability of patients to obtain care when they need; (2) relationship between the patient and health professionals: ability to generate motivation in the patient so that the approach of the health team is effective (this is the most sensitive indicator to reflect the success or failure of the care outcome); (3) conditions of the structure of the service: ability to generate privacy, comfort, cleanliness, and other conditions that show the respect for the patient; (4) information to the patient: ability to inform patients especially regarding the effects, risks, and costs of treatment, considering their values, expectations, and opinions; and (5) equity and opinion of the patient: ability to consider the opinion of the patient regarding what is fair and equitable, which includes general subjective aspects related to the individual, sometimes different from what is socially expected^6^ ^,^ ^7^. According to Bolzan^2^, the OAS is one of the instruments with the greatest potential to capture effectively the characteristics of acceptability.

The perception regarding health practices and user satisfaction is a notorious subject in the health scenario in the world and national context. Evaluations represent a way to seek improvement in the quality of health services and to create opportunities to discuss the patient care^8–10^.

The *Cegonha* Network is a program instituted in 2011, with Ordinance 1,459 of the Ministry of Health. It aims to establish a model of delivery care that ensures qualified and humanized care for pregnant women, mothers, and children up to two years of age, one of the vital care lines for SUS legitimization^11^. Thus, the objective of this study was to evaluate the implementation of the OAS as an instrument to evaluate the quality of the delivery and birth care in the *Cegonha* Network of the Federal District (DF) of Brazil.

## METHODS

This is a retrospective study, which analyzed data from the telephone survey conducted by the Health Ombudsman service of the Health Department of the Federal District (SES-DF), Brazil. The survey interviewed mothers with deliveries between October 15, 2013 and November 19, 2013, in the 12 public maternity hospitals that make up the *Cegonha* Network of SES-DF, namely: Hospital Regional da Asa Norte, Hospital Materno Infantil de Brasília, Hospital Regional de Brazlândia, Hospital Regional de Ceilândia, Hospital Regional do Gama, Hospital Regional do Paranoá, Hospital Regional do Planaltina, Hospital Regional de Samambaia, Hospital Regional de Santa Maria, Hospital Regional de Sobradinho, Hospital Regional de Taguatinga, and Casa de Parto de São Sebastião.

**Figure 1:**
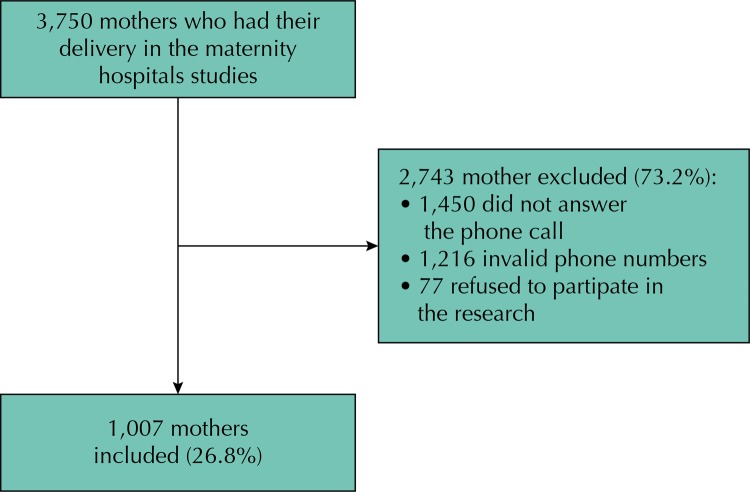
Inclusion flowchart. *Cegonha* Network of Federal District, Brazil, October to November, 2013.

There were 3,750 births in the maternity hospitals during the period. The inclusion criteria were: parturients who provided valid telephone numbers, who answered the telephone call, and who accepted to participate in the evaluation research. Thus, 1,007 parturients were included in the study (26.8%) (Figure 1).

The predominant age groups were 25 to 29 years (26.3%) and 20 to 24 years (24.2%). Half of the women had up to High School Education (50.1%), 29.4% had up to Basic Education II, and 13.0% had up to Basic Education I. The place of residence was the DF for 69.7% of the interviewees and the region surrounding the DF belonging to the state of Goiás for 4.7% of them. Most births occurred in the maternity hospitals of Hospital Regional de Ceilândia (17.6%), Hospital Materno Infantil de Brasília (16.8%), and Hospital Regional de Santa Maria (14.4%) (Table 1).

**Table 1:** Characteristics of the parturients who answered the questionnaire. *Cegonha* Network of Federal District, Brazil, October to November, 2013.

Variable	n	%
Age group (years)		
10 to 14	4	0.4
15 to 19	205	20.4
20 to 24	244	24.2
25 to 29	265	26.3
30 to 34	184	18.3
35 to 39	81	8.0
40 to 44	21	2.1
45 to 49	2	0.2
Above 50 years	1	0.1
Education level		
Illiterate	5	0.5
Basic education I (up to 5th year)	131	13.0
Basic education II (up to 9th year)	296	29.4
High school education	505	50.1
Undergraduate education	67	6.7
Graduate education	3	0.3
Place of residence		
Federal District	702	69.7
Region of the Surrounding Area belonging to Goiás	249	24.7
Other States	56	5.6
Maternity hospital of the delivery		
Hospital Regional de Ceilândia	177	17.6
Hospital Regional da Asa Sul	169	16.8
Hospital Regional de Santa Maria	143	14.2
Hospital Regional do Gama	113	11.2
Hospital Regional de Sobradinho	93	9.2
Hospital Regional do Paranoá	66	6.6
Hospital Regional de Samambaia	59	5.9
Hospital Regional de Brazlândia	54	5.4
Hospital Regional de Taguatinga	51	5.1
Hospital Regional de Planaltina	42	4.2
Hospital Regional da Asa Norte	25	2.5
Casa de Parto de São Sebastião	15	1.5

Total	1,007	

In order to recruit the sample, each parturient was individually invited to participate in the research, with the personal visit of a member of the Health Ombudsman service of the SES-DF during hospitalization at the maternity hospital, when an invitation card was given. The questionnaire of acceptance evaluation was applied by telephone interview approximately on the 15th day after delivery.

The research instrument was a questionnaire prepared by the Central Ombudsman service of the SES-DF and the Coordination of Scientific Research and Communication of the Escola Superior de Ciências da Saúde, Brasília, DF, Brazil. The instrument has 25 closed multiple choice or Likert scale questions (very poor, poor, fair, good, and excellent), which includes sociodemographic data and evaluation of the acceptability in its five domains: (1) accessibility – eight questions; (2) relationship between the patient and health professionals – two questions; (3) conditions of the structure of the service – five questions; (4) information to the patient – three questions; and (5) equity and opinion of the patient – three questions. Some questions in the domain of accessibility were transcribed from the Preliminary Research Report of the SUS General Ombudsman service, Ministry of Health, Brazil^12^.

We studied the qualitative or categorical variables according to the frequency and distribution of proportions. We used the score transformed into a scale from zero to 100, zero related to the worst evaluation (very poor) and 100 to the best evaluation (excellent) for the analysis of Likert-type scale questions. The results were expressed as mean and standard deviation^10^.

The study is part of the research project Organization, Access, and Continuity of Care in the Maternal and Child Health Network, of the Federal District Health Department, approved by the Health Ethics Committee of the SES-DF in 2015 (Process CAAE 01918712.6.0000.5553).

## RESULTS

Among the 1,007 parturients included in the research, 74.3% of the women had prenatal care exclusively in the DF and 22.4% had it exclusively outside the DF. Approximately 0.2% of the parturients did not have prenatal care and 85.4% had six or more prenatal appointments (Table 2).

**Table 2:** Distribution of the answers of the parturients in relation to the evaluation of accessibility and conditions of the service. *Cegonha* Network of Federal District, Brazil, October to November, 2013.

Variable	n	%
Accessibility		

Place of prenatal follow-up		
Exclusively in the Federal District	748	74.3
Partially in the Federal District	27	2.7
Exclusively in other states	226	22.4
No prenatal care	6	0.6
Number of prenatal appointments		
Zero	2	0.2
1	2	0.2
2 to 3	40	3.9
4 to 5	104	10.2
6 to 7	214	21.0
Above 7	869	64.4
Waiting time for the initial care of the health team before admission to the Obstetric Center		
Immediate	339	33.7
Up to 30 minutes	253	25.1
Above 30 to 1 hour	142	14.1
Above 1 hour up to 2 hours	91	9.0
Above 2 hours up to 3 hours	40	4.0
Above 3 hours up to 4 hours	28	2.8
Above 4 hours	111	11.0
Does not remember	3	0.3
Delivery carried out directly in the first maternity hospital sought	801	79.5
Home visit by community health agent after discharge from maternity hospital	184	18.3

Conditions of the service		

Lack or materials		
Bed sheets	279	27.7
Gown	247	24.5
Hand sanitizer	64	6.4
Medications	51	5.1
Toilet paper	36	3.6
Paper towel	35	3.5
Medical devices	29	2.9
Did not happen	608	60.4

**Figure 2:**
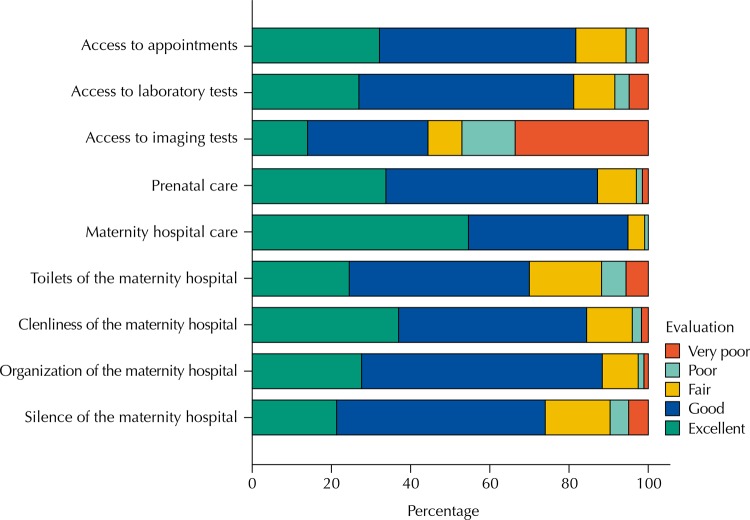
Evaluation of the prenatal and maternity hospital care. *Cegonha* Network of Federal District, Brazil, October to November, 2013.

Among the parturients with exclusive prenatal follow-up in the DF, access to appointments was evaluated as good or excellent by 86.1% of them (score transformed into scale = 76.6±22.8) and access to laboratory tests was evaluated as good or excellent by 85.2% of them (score transformed into scale = 73.9±24.4). Access to the imaging tests had a worse performance: 45.7% of the parturients evaluated it as good or excellent (score transformed into scale = 44.5±38.0) (Figure 2).

Deliveries occurred in the same maternity hospital as the initial care for 79.5% of the women. The waiting time for the initial care provided by the health team before admission to the Obstetric Center was immediate in 33.7% of the answers, up to half an hour in 25.1% of them, and up to one hour in 14.1% of them. Among parturients who waited for more than one hour, waiting time was above five hours in 11.0% of the deliveries (n = 111/1,007) (Table 2).

Approximately 18.3% of the mothers received home visits after discharge from the maternity hospital (Table 2).

The reception and cordiality received during the prenatal care were considered as good or excellent by 85.9% of the women (score transformed into scale = 79.3±22.2) and the quality of the maternity hospital was classified as good or excellent by 94.8% of them (n = 955/1,007) (score transformed into a scale = 87.1±15.9) (Figure 2).

The evaluation about the silence of the environment was excellent or good in 74.0% of the occasions (score transformed into scale = 70.2±24.6). The organization of the environment was reported as excellent or good by 88.5% of the women (score transformed into scale = 78.1±17.1). The evaluation about the cleanliness of the maternity hospital was excellent or good for 84.5% of the parturients (score transformed into scale = 78.9±21.0). The condition of the toilets was reported as excellent or good in 70.0% of the answers (score transformed into scale = 69.3±26.5) (Figure 2).

The main items perceived as lacking during the stay in the maternity hospital were bed sheets (27.7%), gown (24.5%), and hand sanitizer (6.4%). However, most (60.4%) did not perceive a lack of items during their stay in the maternity hospital (Table 2).

Of the women interviewed, 76.3% reported having been informed by the health team about the date and Health Center to where they should take the newborn for the first appointment, and 94.8% received guidance and help with the practice of breastfeeding still in maternity (n = 955/1,007). However, only 41.0% of the women were informed in the prenatal period about the maternity hospital where they should go for the delivery (Table 3).

**Table 3:** Distribution of the answers of the parturients in relation to the evaluations on information received, equity, and opinions considered. *Cegonha* Network of Federal District, Brazil, October to November, 2013.

Variable	n	%
Information received		
Prenatal information on the maternity hospital where the child should be delivered	413	41.0
Guidance and help on the practice of breastfeeding in the maternity hospital	955	94.8
Information regarding the date and Health Center where the newborn should be brought for the first appointment	768	76.3
Equity and opinions considered		
Newborns placed skin-to-skin immediately after birth	695	69.0
Companion of choice at delivery	492	48,9
Occurrence of disrespectful situation		
Bad service	67	6.7
Not answered/heard in her need	26	2,6
Verbal aggression	26	2,6
Physical aggression	3	0.2
Did not happen	887	88.1

Most respondents reported that the newborns were placed skin-to-skin immediately after birth (69.0%, n = 695/1,007). However, only 48.9% had a companion of their choice at the time of delivery (Table 3). Of the parturients, 7.5% were not advised as to their right to have a companion. The presence of a companion was denied in 8.6% of the cases.

Although 88.1% of the interviewees reported that they did not go through disrespectful situations, there were situations understood by the parturients as verbal aggressions in 2.6% of the cases and as physical aggressions in 0.2% of the cases. In addition, 9.2% reported that they were poorly cared for or that their needs were not met/listened to (Table 3).

## DISCUSSION

In this study, some of the good obstetrical practices were mentioned by less than half the respondents, such as information regarding the maternity hospital where the child should be delivered and the presence of a companion of their choice at the time of delivery. The proportion was also high for respondents who reported a lack of supplies and who had no visit from a community health agent (CHA) after discharge. Nevertheless, the sums of the good and excellent answers are above 80% in the evaluation of the satisfaction with access to prenatal care, laboratory tests, and reception and cordiality in the prenatal care and maternity hospital. These results should be evaluated critically, especially in relation to the possibility of gratitude or courtesy bias^9^ ^,^ ^10^ ^,^ ^12^. Similar results have been found in a study that has evaluated abortion care in maternity hospitals in the Brazilian Northeast, in which respectful treatment was the best well evaluated, despite the inadequacy of fundamental aspects of the care, such as pain relief, guidelines about the care, post-discharge review, and the infrastructure of the units^13^.

The evaluations of user satisfaction came from the theoretical frameworks of marketing and social psychology^14^. Among the theoretical models, the discrepancy theory is the most commonly used proposal. In this theory, satisfaction levels are measured by the difference between expectation and perception of experience. However, recent studies have shown that, on some occasions, high levels of user satisfaction can be reported, which are dissociated from the actual quality of health services used^10^ ^,^ ^12^. Explanations for this paradox can be attributed to lack of information, poor expectation of the service, and possible biases, such as courtesy or gratitude, which hinder a critical view about the service^9^ ^,^ ^12^.

The OAS worked as an innovative device in the evaluation of the acceptability of the *Cegonha* Network of the DF. We could obtain information from the five sub-dimensions of acceptability as defined by Donabedian^5–7^. This is a fundamental aspect, especially in the evaluation of a program in which it is fundamental the access to health practices based on the best scientific evidence and the recognition of the pregnant woman and her family members as central players, rather than mere spectators in the maternal and child care^15^ ^,^ ^16^.

In relation to primary health care, there was good prenatal care in the *Cegonha* Network of the DF. Only two parturients did not have any prenatal appointment. This result is above that reported for the Brazilian Midwest region in a study that has evaluated the prenatal care offered to pregnant women in public or private health services in Brazil from 2011 to 2012^17^. The percentage of pregnant women with at least six appointments, as recommended by the Ministry of Health^11^, was above that observed in previous studies^18–24^.

An important aspect to be analyzed is the link between parturients and maternity hospital of reference. Although there was a low rate of pilgrimage among pregnant women in search of maternity hospitals, as most had their delivery in the first maternity hospital sought and received care within one hour, less than half received information in the prenatal care about the maternity hospital that they should look for at the time of the delivery. Moreover, one-third of the deliveries performed in maternity hospitals of the DF were for women living in other Brazilian States. Therefore, there was no good link between the parturient and her maternity hospital of reference. This shows that the maternity hospital-basic health unit-community health agent (MAT-UBS-ACS) reference, which is a key point in ensuring the integrality of the maternal-neonatal care, remains ineffective. This highlights the still insufficient role of the prenatal care in preparing pregnant women for delivery, as observed in other studies in Brazil^25–28^.

Another factor draws our attention for this low effectiveness of the MAT-UBS-ACS reference. Most women received information about breastfeeding and the health center where they should go for the first appointments after discharge from the maternity hospital. However, few reported a home visit of a community health agent until the interview, which occurred approximately on the 15th day after delivery.

The structure of the health services and the care of the health professionals (cordiality and reception) were well evaluated. Similar results have been found in studies with mothers in Chilean and Brazilian maternity hospitals, which highlights the relevance of the relationship between professionals and patients in parturient/mother satisfaction^29^ ^,^ ^30^.

Approximately 40% of the women reported lack of materials during hospitalization, mainly personal items, such as bed sheets and gown. In addition, aspects related to the humanization in the delivery room need to improved, such as the right to a companion and the attitude of the skin-to-skin contact of the newborn immediately after birth. These practices are recognized as beneficial for childbirth care with several favorable effects. Reports of disrespectful situations or aggressions remain, which should not occur under any circumstances. These factors show the need for institutional culture changes, such as the institution of the presence of companions for all women^18–30^.

Access to prenatal appointments and laboratory tests in the prenatal period was rated as good or excellent by most women. Access to ultrasound scans had the worst performance, and less than half of the parturients rated it as excellent or good. This shows the need for managers to act on the qualification of this service. Gestational ultrasound carries a high symbolic value of social nature, considered by pregnant women as an indispensable test and the main technology in the monitoring of pregnancy. This great desire for ultrasounds by the pregnant women may have influenced the low evaluation of the access^31^ ^,^ ^32^.

It is also important to consider the impact of the actions of Health Ombudsman services in the Management process. In this study, we highlighted fundamental elements to improve the “know-how”. Classically, the response time is measured between the complaint/problem, passively received by the Health Ombudsman service, and the referral or solution. The defined work process is: (1) reception, (2) analysis, (3) forwarding, (4) follow-up, (5) answer, and (6) recording^33^. Similarly, the National Health Surveillance Agency (ANVISA) reinforces the vision of the Ombudsman service as a consumer defense, mediator of conflicts and fundamental to improve participatory democracy. It also uses it as an indicator of effectiveness – the number of demands received versus the number of demands met, focusing on health surveillance. The brand of excellence was defined in a management agreement between ANVISA and the Ministry of Health, when the deadline of 15 working days to answer 80% of the messages received was established^34^. In both references, the strategic objective is the improvement of the quality of the SUS. However, the proposal of the Ombudsman service established by the SES-DF, called the Active Ombudsman Service, has the objective of acting in the active collection of information to subsidize effectively the managers and users of the SUS for faster and more efficient decision-making. In this way, the social participation and the ombudsman service are strengthened, as a space of collective listening for social control and monitoring of health actions and services offered by the SUS. Therefore, it is not a matter of referring a complaint or problem but verifying the strategic alignment of the service provided with the management levels of the SUS-DF.

One limitation of the study is its descriptive characteristic. Because this was a convenience sample that included only the parturients who consented to perform the telephone interview, there may have been selection biases. Moreover, no analysis was performed to identify whether there was a relationship between the worse evaluation in the indicators and the characteristics of the women, such as education level and place of residence. However, given the importance of the subject, we can reflect about the possibility of success of the fundamental objective of Health Ombudsman services, which is to give voice to the users in the management process. The results of this research can support actions to improve the quality of services insofar as they point out the main aspects of the *Cegonha* Network that are not adequate in the region. The results can also be used as a baseline for subsequent evaluations on gestation, delivery, and birth care and for the improvement of the OAS device as a strategy to include citizens in the process to qualify the SUS.

This paper corroborates the role of the OAS as an innovative device to evaluate the quality of the health care. It promotes the incorporation of the perspective of the female user in the evaluation of the acceptability of the *Cegonha* Network of the DF, which may contribute in decision making processes for the improvement of health services.
